# Alternative mating strategies in the Wellington tree weta expressed via genetic polymorphism for precocial male maturation

**DOI:** 10.1098/rsos.240100

**Published:** 2024-02-21

**Authors:** Clint D. Kelly

**Affiliations:** Département des Sciences biologiques, Université du Québec à Montréal, Montreal, Quebec, Canada H3C 3P8

**Keywords:** alternative mating strategy, genetic polymorphism, Mendelian strategy, precocial

## Abstract

The expression of alternative mating strategies is generally hypothesized to be a plastic response of the genome to the environment or a genetic polymorphism. The Wellington tree weta (*Hemideina crassidens*) is an orthopteran insect in which males express three alternative mating morphotypes related to the size of mandibular weaponry. My common garden experiment in the laboratory shows that male weapon size at maturity in this species is nether condition- nor environment-dependent; therefore, supporting the hypothesis that it is genetically polymorphic. Males that matured at the 8th instar had the shortest development time and the smallest weaponry, males that matured at the 10th instar had the longest development time and the largest weaponry, and males that matured at the 9th instar were intermediate to the other two morphs. These morph-related differences in development time suggest that male alternative mating strategies in this species are expressed via precocial male maturation, a rare mode of strategy expression in insects. Additionally, adult lifespans differed significantly among morphs with 8th instar males having the shortest, and 9th instar males having the longest lifespans. I discuss how these differences in lifespan align with morph-specific investment in pre- and post-copulatory processes to equalize fitnesses among morphs.

## 1. Introduction

Alternative mating strategies are taxonomically widespread across animals with conventional individuals (typically males) typically being dominant, territorial, and possessing weaponry and/or courting females, whereas unconventional males are subordinate, non-territorial, have reduced or no weaponry, and adopt sneak mating tactics to achieve copulation [1–3]. There is little argument that intense sexual selection is ultimately responsible for the evolution of alternative mating strategies but there is much less consensus as to how these alternatives are expressed [1–5].

One hypothesis posits that male alternative phenotypes are plastic responses of the genome to the environment or to maternal effects and are underpinned by polygenic, condition-sensitive regulatory mechanisms [1,5,6]. In other words, alternative phenotypes could be produced by G × E interactions in which individuals express morphotypes based on a switchpoint or threshold and the prevailing environmental conditions; individuals with genotypes below a threshold express one phenotypic character state while those with genotypes above the threshold express a modified or alternative state [6,7]. For example, poorly fed individuals are expected to adopt the unconventional phenotype (below the threshold) while well-fed individuals express the conventional phenotype (above the threshold) [6]. This hypothesis assumes that the threshold position is genetically variable and heritable [1,5,6]. Although most alternative mating strategies across animal taxa, including insects [8], appear to be conditional (or developmental), whether these alternative morphotypes must have equal fitness to be maintained in a natural population remains a matter of debate [1,2,4–6,9]. Alternatively, mating strategies could be genetically polymorphic, whereby the different morphotypes are influenced by a few loci of major effect, and males exhibit within-population Mendelian segregation [1,6]. In these cases, male nutritional condition (or any status-related trait like age or health) is not relevant to morph expression. This hypothesis predicts that strategies have equal fitness [1,6]. Confirmed cases of genetic polymorphisms are scarcer than conditional or developmental strategies and include the ruff *Philomachus pugnax* [10], the side-blotched lizard *Uta stansburiana* [11], an acarid mite [12], the marine isopod *Paracerceis sculpta* [13], fig wasps [14,15], poeciliid fishes [16–18] and a damselfly [19].

Unconventional males typically lack exaggerated weaponry for combat with rivals or showy ornaments for attracting females [1,3]. The most common mechanism used by insects to achieve an irreversible unconventional phenotype is condition-dependence with expression dependent on, for example, larval size or condition; poor nutritional condition results in small body size and small (or no) secondary sexual characters such as weaponry. In scarab horned beetles, for example, adult male body and weapon size (i.e. morphotype) appears to depend on larval mass [20–22]. Another way in which males can achieve such a stripped-down, alternative phenotype is via precocial maturation [1]. Precocial maturation (or heterochrony) occurs when males in a population sexually mature (i.e. have functional testes) early along their growth trajectory before acquiring secondary sexual characters (i.e. weapons and ornaments). Because precocial (unconventional) males lack exaggerated secondary sexual traits they should use mating tactics that differ from those adopted by conventional males. Precocial male maturation is not to be confused with protandry whereby males emerge, on average, earlier than females because this gives them a mating advantage over later-emerging rivals [23]. In protandrous species, early emerging males, compared with later-emerging conspecifics, generally complete the same developmental programme but only quicker (i.e. have same number of instars), have the same morphology and secondary sexual characters (but with perhaps smaller body size), and use the same mating tactics to obtain copulations [24,25]. That is, males having different emergence times in protandrous species do not exhibit alternative mating strategies nor do they adopt alternative mating tactics.

Precocial maturation is taxonomically widespread [5,26] and can occur in insects through the elimination of instars; however, instar number is generally canalized across insect species [24]. If there is variation in instar number within a species, the difference is usually between males and females (with females having more instars) and rarely is the variation intrasexual [27]. Plasticity in instar number can be caused by several environmental factors including temperature, photoperiod, rearing density, and food quality and availability [24,27]. To my knowledge, there is no evidence from a natural insect population for variable instar numbers within a sex to be driven by a genetic polymorphism, nor do I know of any insect species in which alternating mating strategies are a product of precocial male maturation. It also appears that early maturation by males as an alternative mating strategy has been identified in fishes only [1] and in some of these cases early maturing males can eventually reach a more competitive size because they continue to grow after sexual maturity [28], which is not the case in insects. Whether the limited taxonomic diversity of males adopting precocial maturation as an alternative mating strategy is owing to it being a rare mechanism in nature or to biased taxonomic sampling is unclear. That said, it is indeed remarkable that precocial male maturation has not been documented in an insect given that insects express nearly every type of alternative mating phenotype, strategy and tactic observed in nature [8]. Precocial male maturation is unlikely to evolve as a mating strategy in holometabolous insects because juveniles are obligated to pass through distinct developmental stages (i.e. larva, pupa) on their way to adulthood; however, it is possible that larvae could adjust their number of instars prior to pupation but data on whether this occurs are not available. It is surprising, however, that alternative strategy expression via precocial male maturation has not been documented in a hemimetabolous insect. This developmental system seems ideally suited to invade a population of conventional males since juveniles are just smaller versions of adults that accrue body size with each instar until maturity, and it is possible in many species to add or reduce instars before sexual maturation [29].

Given the paucity of precocial male maturation-derived mating strategies in insects, it is not surprising that we know nothing about morph-specific life-histories of such strategies and how they might influence fitness equalization across morphs. For example, a simple scenario suggests that if total lifespan (i.e. hatching to death) is similar among all morphs then development time (time as a juvenile) will trade off with adult longevity (and vice versa), meaning that although (early maturing) unconventional males might have poorer daily mating success, on average, than their conventional counterparts, their longer reproductive lifespan allows them to accrue lifetime mating success and fitness equal to (later-maturing) conventional males, all else being equal [19]. Alternatively, if unconventional males prolong the duration of instars, that is development time is similar among morphs, as is adult lifespan (i.e. equal total lifespans), then unconventional males might compensate for their lower mating success by increasing their fertilization success via, for example, superior sperm competitiveness. A first step in deciphering how life history could explain fitness equalization among morphs is to measure development time and adult lifespan for each morph.

The Wellington tree weta (*Hemideina crassidens*) is a flightless and hemimetabolous orthopteran insect that is endemic to New Zealand [30]. Both sexes seek diurnal safety from predators in tree cavities, and because these cavities can be limiting in nature, several weta can simultaneously reside in them [31–34]. This scenario creates an opportunity for harem formation, which in turn provides males with an opportunity to increase their reproductive success by monopolizing females. Indeed, male *H. crassidens* use their enlarged mandibles in combat with other males for control of female-occupied galleries [31,32,35,36], and males with larger mandibles enjoy greater harem success [33,37], probably because larger mandibles are superior weapons [38]. Strong sexual selection, however, has apparently favoured the evolution of two alternative strategies in this species ([39]; see also [40]). Some males possess small bodies and small weaponry. These males appear to circumvent the defences of larger males and sneak copulations or search the foliage for undefended females. The largest males have significantly larger weaponry and greater resource-holding potential than smaller males and consequently tend to reside with larger groups of females. An intermediate morph appears to be a jack-of-all-trades that can sneak or fight depending on the situation. Males are hypothesized to sexually mature at different instars with small and intermediate males maturing precocially at the 8th and 9th instars, respectively, whereas the largest males mature at the 10th instar, similar to females [40]. How males express these different morphotypes is not known; however, they bear the hallmarks of being controlled by a genetic polymorphism, given that the three male morphs persist in stable proportions across time [39], suggesting that morph frequency in this population is at or near equilibrium, and the strategies have equal fitness [41].

In this study, I use a common garden experiment to test the hypothesis that alternative strategy expression in *H. crassidens* is controlled by a genetic polymorphism for precocial maturation. I predict that if male morphotype is dictated by a genetic polymorphism then males will express different morphotypes by maturing at different instars despite experiencing the same environmental conditions during development (see [42]). I also examine between-sex and among-morph differences in development time and adult lifespan to explore the role that life history plays in equalizing fitnesses among morphs.

## 2. Methods

I haphazardly collected 35 adult female tree weta at night from vegetation on Maud Island, New Zealand in March 2015. Females were brought into the laboratory and placed in a 5 l pail having a lid with holes for aeration. Each pail contained an artificial wooden cavity for diurnal refuge, a container of moist vermiculite for oviposition, and a piece of apple and dried cat food to provide food and water. Females were checked every other day and food was replenished as needed. Females were left to oviposit for two weeks, after which they were released back into the wild at their point of capture.

I sifted the oviposition substrate for eggs. The approximately 200 eggs were haphazardly placed in several 1.5 ml microbes and returned to the laboratory in Montreal, Québec, Canada where they were placed in groups of 10 on moist vermiculite in 250 ml deli containers. Eggs were covered with a thin layer of moist vermiculate and a lid was placed on the container. Maternal identity of eggs was not recorded. Containers were placed in an incubator (Percival I-41VL, Perry, IA, USA) whose temperature and light regime were set to follow those of Wellington, NZ from April to December (mean monthly temperatures and light regime). Vermiculite was remoistened and fungus removed if necessary during weekly inspections of the containers.

Beginning mid-October, containers were inspected daily for hatchlings. When a hatchling was discovered it was removed from the oviposition container, its hatch date was recorded, it was assigned an alphanumeric ID, and its dorsum was digitally photographed using a Leica SC170 HD camera (Leica Microsystems Inc., Concord, ON, Canada) connected to a Leica S6D microscope (Leica Microsystems Inc., Concord, ON, Canada). We used LAS v. 4.5.5 software (Leica Application Suite) to digitally stamp a scale bar on images. We then imported each image into ImageJ (National Institutes of Health, USA) to measure pronotum length (a proxy for body size in tree weta [43].

Newly hatched individuals were then individually placed in a new 250 ml container and fed ad libitum with fish food (Tetra Flakes) and a small piece of apple and provisioned with a cotton-plugged 0.5 ml microcentrifuge tube filled with water. Containers were checked weekly, and food and water were replenished as needed. Weta (*n* = 61 females; *n* = 64 males) were photographed as above approximately every 21 d until eclosion to adulthood. Weta were transferred to larger containers (12.1 l, IRIS Airtight Pet Food Container, Pleasant Prairie, WI, USA) three months after hatching. These containers contained a wooden cavity for refuge, a large piece of egg carton for climbing, and a cotton-plugged Falcon tube (50 ml) filled with water. The lid of the container contained a mesh-covered hole for aeration as well as to provide purchase for moulting. The weta's diet of fish food and apple was then expanded to include organic spinach, bee pollen, dried cat food, frozen crickets and carrot. Containers were cleaned and food changed three times per week. The date of opportunistic observations of moulting in progress or evidence of freshly moulted exuviae were recorded. The date on which weta eclosed to adulthood was recorded and head length (a proxy for weapon length) was measured to the nearest 0.01 mm by using digital callipers (Fisher Scientific, St Laurent, Quebec, Canada) (see [37]). All juvenile tree weta were raised individually and experienced identical rearing conditions from egg to adulthood. Adults were also raised individually and observed until their death at which time the date was recorded.

### 2.1. Statistical analysis

All statistical analyses were conducted in the R statistical environment [44].

I hypothesized that males reared in a common garden would exhibit trimorphism if their alternative phenotypes were the result of a genetic polymorphism for precocial maturation. I tested this hypothesis by first determining the number of instars required by each weta (*n* = 61 females; *n* = 64 males) to reach adulthood by examining a plot of pronotum length (mm) on measurement date and counting the jumps in growth. Opportunistic observations of moulting were also used to corroborate plot-derived growth increments. I then tested the predictions that males would have larger heads than females and that head length would positively correlate with final instar in males by using a generalized linear model with head length (mm) as the response variable and sex/male instar as the independent fixed factor.

Previous studies concluded that male *H. crassidens* are trimorphic with respect to head length (i.e. weapon length) in the wild, based on maximum-likelihood analysis of mixture models (see McLachlan and Basford [45] for detailed explanation of likelihood analyses of mixture models) that better supported a head-length distribution of three modes rather than two or four modes [37,39]. Following Kelly and Adams [39], I corroborated that approach here by testing whether head length distribution of laboratory-reared males is best described as comprising three modes. I first used the R package *mixsmsn* to generate a bimodal model and a trimodal model containing distributions corresponding to two and three modes, respectively. I then compared each model's Akaike information criteria (AIC), calculated as 2*k* – 2ln(*l*), where *k* is the number of model parameters, and ln(*l*) is the natural log of the maximized value of the model's likelihood function. The model with the lowest AIC was accepted as the more parsimonious model. I then compared the AIC of a trimodal model with that of a tetramodal model, with the model having the lowest AIC deemed most parsimonious. The mixture model analyses used all laboratory-reared tree weta (*n* = 74 females; *n* = 84 males) independent of whether developmental history was known or the duration of life-history stages was recorded.

I tested differences between the sexes and among morphs in development time (hatch to eclosion to adulthood), adult longevity (eclosion to death), and total lifespan (hatching to death) by using a generalized linear model with Poisson family of errors. For each model, time (days) was entered as the response variable and sex/male instar was entered as the independent fixed factor. The time required to eclose to adulthood was recorded for *n* = 61 females and *n* = 64 males whereas adult longevity was recorded for *n* = 58 females and *n* = 63 males.

For all generalized linear models, I constructed planned contrasts in which females were contrasted with males (combined 8th, 9th and 10th instar males). For models testing morph differences in development time and total longevity, 8th instar males were contrasted with 9th instar males, and 9th instar males were contrasted with 10th instar males. Visual inspection of box plots comparing adult lifespan among male morphs, however, suggested that 9th instar males had the longest duration of the three. I therefore contrasted 8th instar with 10th instar males and 9th instar with 10th instar males. If 9th instars have longer lives than 10th instars, which in turn have longer lives than 8th instars, then 9th instar must have longer lives than 8th instars. Model assumptions of normally distributed residuals and homogeneity of variances were tested by using the *check_model* function in the R package *performance* [46]. Means are presented ± one standard error of the mean unless otherwise noted.

## 3. Results

**Figure 1.**
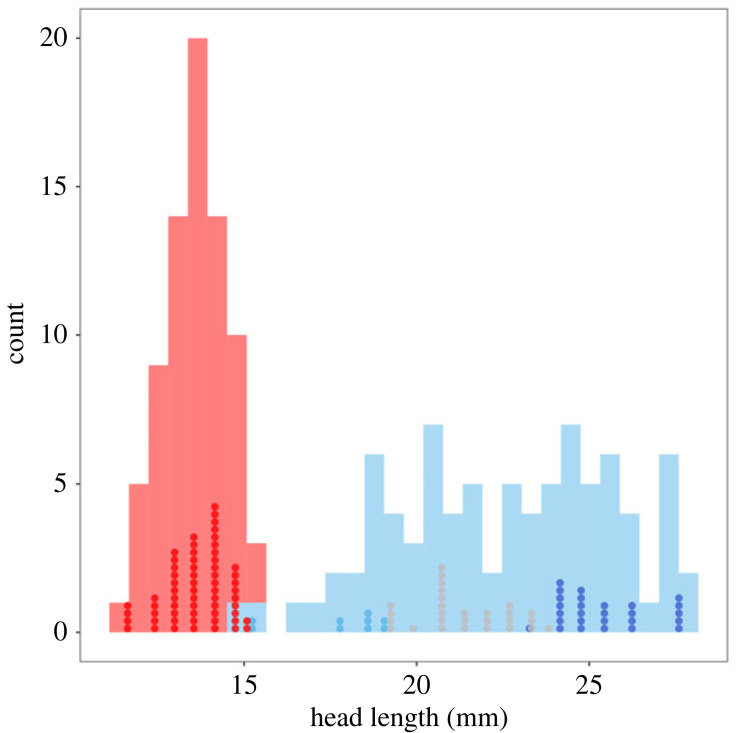
Head length frequency distribution for laboratory-reared female (red; *n* = 74) and male (blue; *n* = 84) *Hemideina crassidens* tree weta. Dots represent head length distribution of subset of tree weta for which instar at maturity is known (females, red; 8th instar males, light blue; 9th instar males, grey; 10th instar males, dark blue).

Males in my common garden experiment whose development was tracked through time (*n* = 63) revealed that they matured at either the 8th, 9th or 10th instar, thus supporting the hypothesis that male weapon trimorphism is a genetic strategy in this species (figure 1). By contrast, females matured at the 10th instar only. As predicted, a mixture model analysing the full dataset of males (*n* = 84) better supported a model predicting three morphs than a model predicting either two (AIC_2_ = 433.33; AIC_3_ = 425.72; ΔAIC = 7.61) or four (AIC_3_ = 425.72; AIC_4_ = 431.65; ΔAIC = 5.93) morphs (figure 1). By contrast, a single distribution was better supported in females as compared with a bimodal distribution (AIC_1_ = 195.08; AIC_2_ = 198.92; ΔAIC = 3.85) (figure 1).

As expected, I found very strong evidence that mean head length was longer in males ($\overset{↼}{x}=22.59\pm 0.38\;\mathrm{mm}$, *n* = 63) than in females ($\overset{↼}{x}=13.61\pm 0.12\;\mathrm{mm}$, *n* = 60; estimate = 5.937 ± 0.169, *z* = 35.16, *p* < 0.001) and within males, 8th instar males ($\overset{↼}{x}=17.79\pm 0.51\;\mathrm{mm}$, *n* = 9) had smaller heads, on average, than 9th instar males ($\overset{↼}{x}=21.36\pm 0.27\;\mathrm{mm}$, *n* = 27; estimate = −3.732 ± 0.280, *z* = −13.33, *p* < 0.001) who in turn had significantly smaller heads, on average, than 10th instar males ($\overset{↼}{x}=25.43\pm 0.25\;\mathrm{mm}$, *n* = 27; estimate = −3.899 ± 0.212, *z* = −18.41, *p* < 0.001).

**Figure 2.**
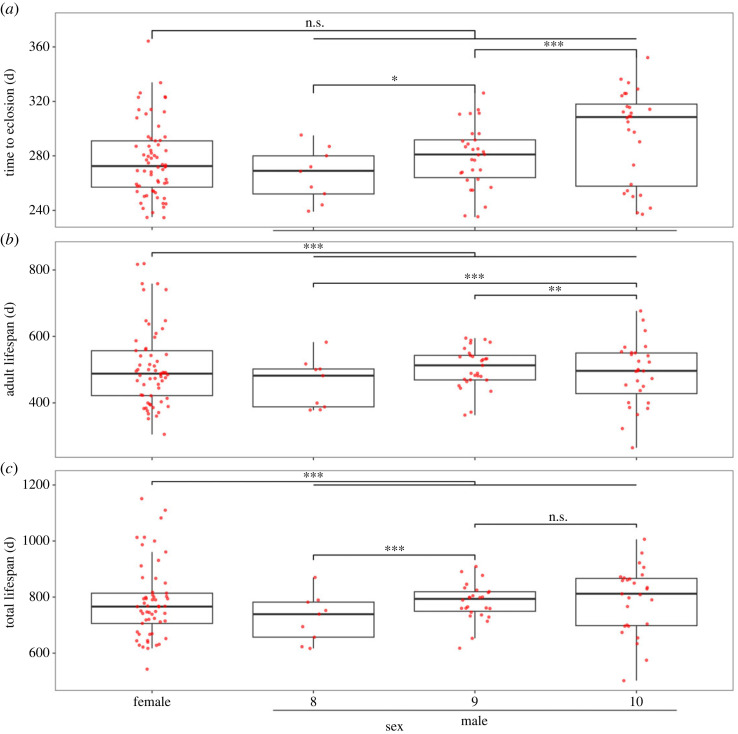
(*a*) Time to eclosion (days) (*b*) adult lifespan (days) and (*c*) total lifespan (hatch to death, days) of laboratory-reared female and male *Hemideina crassidens* tree weta. *p < 0.05, **p < 0.01, ***p < 0.001; n.s. = non-significant.

I found weak evidence that the sexes differed in their time to eclosion (females: $\overset{↼}{x}=274.95\pm 3.47\;d$, *n* = 60; males: $\overset{↼}{x}=284.97\pm 3.68\;d$, *n* = 63; estimate = 0.015 ± 0.009, *z* = 1.71, *p* = 0.086; figure 2*a*). Male morphs, however, differed in development time with 8th instars ($\overset{↼}{x}=266.11\pm 6.47\;d$, *n* = 9) maturing faster than 9th instars ($\overset{↼}{x}=279.10\pm 4.21\;d$, *n* = 30; estimate = −0.053 ± 0.015, *z* = −3.60, *p* < 0.001) and 9th instars maturing faster than 10th instars ($\overset{↼}{x}=295.32\pm 6.46\;d$, *n* = 28; estimate = −0.057 ± 0.011, *z* = −5.30, *p* < 0.001).

There was very strong evidence that females ($\overset{↼}{x}=507.51\pm 15.79\;d$, *n* = 57) had longer adult lifespans, on average, than males ($\overset{↼}{x}=491.89\pm 10.09\;d$, *n* = 62; estimate = −0.035 ± 0.007, *z* = −5.30, *p* < 0.001; figure 2*b*). The data revealed very strong evidence that male morphs also differed in adult lifespan, with 8th instars ($\overset{↼}{x}=458.67\pm 24.83\;d$, *n* = 9) having shorter adult lifespans, on average, than 10th instars ($\overset{↼}{x}=492.52\pm 18.17\;d$, *n* = 27; estimate = −0.054 ± 0.011, *z* = −4.87, *p* < 0.001), and 9th instars ($\overset{↼}{x}=502.73\pm 12.15\;d$, *n* = 26) having a longer adult lifespan, on average, than 10th instars (estimate = 0.037 ± 0.008, *z* = 4.50, *p* < 0.001). Consequently, 8th instar males had shorter adult lifespans than 9th instar males.

There was very strong evidence that female total lifespan ($\overset{↼}{x}=782.40\pm 17.42\;d$, *n* = 57) was longer, on average, than male total lifespan ($\overset{↼}{x}=777.10\pm 12.21\;d$, *n* = 62; estimate = −0.017 ± 0.005, *z* = −3.18, *p* = 0.001; figure 2*c*). The data revealed very strong evidence that male morphs also differed in total lifespan, with 8th instars ($\overset{↼}{x}=724.78\pm 28.12\;d$, *n* = 9) having shorter lifespans, on average, than 9th instars ($\overset{↼}{x}=782.31\pm 12.94\;d$, *n* = 26; estimate = −0.054 ± 0.009, z = −6.08, *p* < 0.001), and 9th instars having similar lifespans, on average, to 10th instars ($\overset{↼}{x}=789.52\pm 22.92\;d$, *n* = 27; estimate = −0.032 ± 0.007, *z* = −4.80, *p* < 0.001).

## 4. Discussion

Male Wellington tree weta raised in a common garden eclosed at either the 8th, 9th or 10th instar, whereas females eclosed at the 10th instar only. That all weta in my study matured at one of three instars despite being raised in the same environment with respect to abiotic (i.e. light regime, temperature, container size, cavity size, etc.) and biotic (e.g. diet) factors supports the hypothesis that the alternative mating strategies in this species represent a genetic polymorphism. My conclusion that the alternative mating strategies are genetically polymorphic in Wellington tree weta is further strengthened by previous field studies showing that male morphs have equal fitness [41] and are present in equilibrium proportions in nature [39]. My common garden study, however, cannot identify whether the mode of inheritance of a male's morphotype is Mendelian or polygenic; such a determination requires a quantitative genetic sib analysis [42].

The Wellington tree weta is one of a handful of species to express genetically polymorphic alternative mating strategies. Other taxa include the lekking ruff [10], side-blotched lizard [11], acarid mite [12], marine isopod [13], fig wasps [14,15], poeciliid fishes [16–18,47] and a damselfly [19]. Interestingly, however, none of these cases include a strategy in which males mature precocially. In cases in which males precocially mature as part of a mating strategy, it is assumed that all males in a population have the capacity to mature early and survive to reach an older more competitive age [1]. This approach, however, is possible only in species in which individuals continue to grow after sexual maturation (e.g. salmonids [28,48]), and not possible in animals, such as insects, in which growth (and phenotypic change) stops when the individual reaches sexual maturity. I hypothesize that hemimetabolous insects, particularly orthopterans such as *H. crassidens*, are likely, or even constrained, to evolve this strategy because the necessary developmental machinery is generally already in place. That some hemimetabolous insects (e.g. orthopterans) can add or subtract instars to their developmental schedule depending on environmental conditions (e.g. [49–51]) supports this hypothesis. Therefore, if a conventional strategy possesses large weaponry that are used to gain mating access to females and males can invade this mating niche (*sensu* [1]) by expressing smaller (or no) weaponry, then sexually maturing at a developmental stage when weaponry is relatively smaller should be adaptive. That males in my study maturing at the 8th instar possessed significantly smaller head lengths (i.e. weaponry) than 9th instar males, which in turn possessed heads that were smaller than those of 10th instar males (see also [40]) supports the hypothesis that precocial maturation permits unconventional males to circumvent the fighting and harem-defending strategy of conventional males.

I found that the sexes did not significantly differ, on average, in their development time. Among males, however, 8th instars reached adulthood significantly faster (266 d) than 9th instars (279 d), which, in turn, developed significantly faster than 10th instars (295 d). Therefore, the smallest males not only sexually mature at an earlier developmental stage (i.e. 8th instar) but they achieve this significantly sooner than the other morphs. Similarly, the intermediate-sized males sexually mature earlier (i.e. 9th instar) than the larger, late-maturing 10th instar males. This finding supports the long-standing hypothesis that precocial maturation is the mechanism underlying the male trimorphism observed in *H. crassidens* in nature ([37,39]; see also [40]).

Genetically polymorphic strategies in male *Xiphophorus* swordtail fish also differ in the time to reach sexual maturity with smaller, sneaker male *X. multilineatus* reaching adulthood significantly faster than larger, courter males while intermediate males that both court and sneak were intermediate to the other two morphs [52]. Similarly, in *X. nigrensis*, small sneaker males mature quicker than intermediates, which in turn, sexually mature faster than courters [47]. In the marine isopod *Pa. sculpta*, sneaker (γ) males have higher growth rates and thus reach adulthood quicker than harem-defending (α) males whereas female-mimicking (β) males are intermediate to the other two morphs [13].

Faster development times could be adaptive if, for example, it permits earlier entry to the mating pool by unconventional males; earlier entry could be advantageous if there are fewer conventional competitors or more virgin females that are less choosy. However, reaching sexual maturity by growing faster could be costly to adult longevity [53,54]. For example, *Pa. sculpta* exhibits an inverse relationship between growth rate and reproductive tenure [13] since γ (sneaker) males have significantly shorter adult longevities than β (female-mimic) males, which in turn, have significantly shorter adult lifespans than α (harem-guarding) males. By contrast, it might be adaptive for unconventional males to have longer adult lifespans than conventional males because a longer lifespan allows unconventional males to compensate for their lower relative mating success and thus accrue fitness on par with conventional morphs, all else being equal. For example, Tsubaki *et al.* [19] found that clear-winged (sneaker) male *Mnais pruinosa costalis* damselflies lived longer as adults than orange-winged (territorial) morphs, which probably equalizes the reproductive success of unconventional and territorial morphs. This hypothesis suggests that total lifespan (the time between hatching and death) should be similar between the sexes and among male morphs, with individuals trading off development time for adult longevity. My results suggest that this is not occurring in *H. crassidens* as total lifespan differed significantly between the sexes and among the male morphs with females having longer lives than males and 8th instar males having shorter total lifespans than 9th and 10th instar males, which did not significantly differ.

That total lifespans and development times differ significantly between the sexes and among the male morphs suggests that adult lifespans must differ significantly as well. Indeed, I found that male adult lifespans were shorter, on average, than those of females (508 d) and that 8th instar males had significantly shorter adult lifespans (approx 458 d) than either 9th (503 d) or 10th (493 d) instar males, while the reproductive tenures of 9th and 10th males did not significantly differ. My results are similar to Shuster & Wade's [13] finding in *Pa. sculpta* that fast developers have shorter reproductive tenures (i.e. adult lifespans) but contradict those reporting longer reproductive tenures for unconventional males (e.g. [19]). Why do male *H. crassidens* have shorter adult lifespans than females and why do 8th instar males have lower adult survival than the other two morphs? Perhaps male reproductive behaviour (e.g. mate-searching, sneaking and fighting) exposes males to greater risks of predation and parasitic infection than females. Similarly, perhaps 8th instar males have shorter adult lifespans than other morphs because their reproductive behaviour (i.e. searching and sneaking) incurs greater risks of predation or infection. This, however, is unlikely to explain my observations as tree weta were reared in the current study under benign laboratory conditions free of predators and parasites. Despite being maintained in a benign laboratory environment, the captive tree weta in the current study lived as adults for nearly 1.5 years, on average, which is in line with field observations [55]. With respect to the males in my study, perhaps the shorter reproductive tenures of the 8th instar morph is a cost of faster time-to-maturity (*sensu* [53]). Support for this hypothesis is found in the observation that 8th instar males reached maturity most quickly and exhibited the shortest reproductive tenure whereas 9th and 10th instar males required more time to reach maturity but had longer reproductive lifespans. Although we do not know the growth rates of male *H. crassidens* morphs, it seems likely that males have similar growth rates along a common growth trajectory, with 8th, 9th and 10th instars eclosing to adulthood sequentially along this trajectory. If this is the case, reaching sexual maturity sooner would not come at a cost to adult lifespan because growth rates would not differ among morphs. Further study of tree weta growth rates are required to elucidate the proximate mechanism(s) responsible for the observed sex and morph differences in adult longevity.

Independent of the proximate mechanism underlying the adult longevity of *H. crassidens* morphs, it is clear that the observed morph-specific differences in longevities are adaptive, since fitnesses are equal across morphs [41]. The 8th instar males probably compensate for their shorter reproductive tenures by producing significantly larger ejaculates than 10th instar males [56]; therefore, despite having poorer relative mating success than other morphs ([37]; see also [33]), 8th instars can accrue fertilizations at a rate similar to conventional males [41]. The long adult lifespans of 9th instar males might be related to their jack-of-all-trades strategy in which they are inferior to 8th instars in post-copula competition (i.e. ejaculate investment) and inferior to 10th instars in pre-copula competition (i.e. harem success); therefore, they might need more time as adults to accrue reproductive success equal to the other two morphs. Similarly, the shorter adult longevities of γ and β males in *Pa. sculpta* are not costly to fitness, since fitness is equal across all morphs.

My common garden study provides evidence that precocial male maturation is a genetically polymorphic alternative mating strategy in the Wellington tree weta, *H. crassidens*. The earliest-maturing males lack large mandibular weaponry and have shorter lives as adults compared with late-maturing males that have large weaponry. Although the mechanism driving reduced adult longevities is unknown, it is ultimately of little consequence since these males attain fitness equal to the longer-lived males that mature later.

## Data Availability

The data can be accessed at Open Science Framework via (doi:10.17605/OSF.IO/8G76S) [57]; https://osf.io/8g76s/.
